# A proof-of-concept study to investigate the efficacy of heat-inactivated autovaccines in *Mycobacterium caprae* experimentally challenged goats

**DOI:** 10.1038/s41598-022-26683-0

**Published:** 2022-12-22

**Authors:** Cristian Melgarejo, Carles Planas, Alex Cobos, Claudia Arrieta-Villegas, Iker A. Sevilla, Javier Bezos, Xavier Moll, Yvonne Espada, Joseba M. Garrido, Mariano Domingo, Enric Vidal, Bernat Pérez de Val

**Affiliations:** 1 Unitat Mixta d’investigació IRTA-UAB en Sanitat Animal, Centre de Recerca en Sanitat Animal (CReSA), Campus de la Universitat Autònoma de Barcelona (UAB), Bellaterra, Catalonia Spain; 2 IRTA. Programa de Sanitat Animal, Centre de Recerca en Sanitat Animal (CReSA), Campus de la UAB, Bellaterra, Catalonia Spain; 3 Departament de Medicina i Cirurgía Animals, Universitat Autònoma de Barcelona, Bellaterra, Catalonia Spain; 4 Departament de Sanitat i Anatomia Animals, Universitat Autònoma de Barcelona, Bellaterra, Catalonia Spain; 5 Animal Health Department, NEIKER-Instituto Vasco de Investigación y Desarrollo Agrario, Basque Research and Technology Alliance (BRTA). Derio, Bizkaia, Basque Country Spain; 6 Departamento de Sanidad Animal, Facultad de Veterinaria, Universidad Complutense de Madrid, Madrid, Spain; 7 VISAVET Health Surveillance Centre, Universidad Complutense de Madrid, Madrid, Spain; 8 Fundació Hospital Clínic Veterinari, Universitat Autònoma de Barcelona, Bellaterra, Catalonia Spain

**Keywords:** Vaccines, Bacteria, Predictive markers

## Abstract

This study aimed to assess the efficacy of a heat-inactivated *Mycobacterium caprae* (HIMC) vaccine in goats experimentally challenged with the same strain of *M. caprae*. Twenty-one goats were divided into three groups of seven: vaccinated with heat-inactivated *Mycobacterium bovis* (HIMB), with HIMC and unvaccinated. At 7 weeks post-vaccination all animals were endobronchially challenged with *M. caprae*. Blood samples were collected for immunological assays and clinical signs were recorded throughout the experiment. All goats were euthanized at 9 weeks post-challenge. Gross pathological examination, analysis of lung pathology using computed tomography, and bacterial load quantification in pulmonary lymph nodes (LN) by qPCR were carried out. Only HIMC vaccinated goats showed a significant reduction of lung lesions volume and mycobacterial DNA load in LN compared to unvaccinated controls. Both vaccinated groups showed also a significant reduction of the other pathological parameters, an improved clinical outcome and a higher proportion of IFN-γ-producing central memory T cells after vaccination. The results indicated that homologous vaccination of goats with HIMC induced enhanced protection against *M. caprae* challenge by reducing lung pathology and bacterial load compared to the heterologous vaccine (HIMB). Further large-scale trials are necessary to assess the efficacy of autovaccines under field conditions.

## Introduction

Tuberculosis (TB) is an infectious disease caused by *Mycobacterium tuberculosis* complex bacteria that affects humans and a wide range of domestic animals and wildlife. Animal TB is a zoonotic disease that still represents an important public health issue, particularly in middle and low-income regions where it is estimated to cause up to 10% of human TB cases in some countries^1^. Hence, the control and eradication of TB requires a One Health approach.

Domestic goats are particularly susceptible to this disease^2^, which is mainly caused by *Mycobacterium caprae* and *Mycobacterium bovis*^3^. The goat population in Spain was approximately 2.7 million in 2020 FAOSTAT, accessed on 12/07/2022), being the second largest census of the European Union after Greece. Goat herds are not yet subjected to a National eradication program, except for those epidemiologically related with cattle^4^. Caprine TB causes relevant economic losses on the goat industry in endemic areas^3,5,6^ and infected goats pose a risk of infection to other animal species such as cattle or wildlife, and humans^7^.

Alternative approaches to control livestock TB, such as test-and-segregate or vaccination are being prospected when strict test-and-cull strategies are unfeasible for logistic, economic or cultural reasons^8–11^. In some European regions, vaccination can be an ancillary tool for the control of TB in non-bovine domestic and wild animals in areas with a high burden of TB, particularly in those where multi-host maintenance communities have been identified^12^, hampering the success of bovine TB eradication campaigns.

The safety, immunogenicity and efficacy of *M. bovis* Bacillus Calmette-Guérin (BCG), the only vaccine against TB licensed so far for humans, and badgers in the UK, has been evaluated in goats in experimental and natural infection settings^9,13–15^. However, BCG stability in environmental conditions could be limited and an eventual transmission to other livestock or wildlife species cannot be ruled out^16,17^. Therefore, inactivated mycobacterial vaccines have been also assessed in experimentally^18^ and naturally^19,20^ infected goats.

This study was conceived as an autovaccine proof-of-concept to assess the efficacy of a heat-inactivated *M. caprae* (HIMC) vaccine in goats experimentally challenged with the same strain of *M. caprae* used to prepare the vaccine*,* and to compare it to goats vaccinated with a previously characterized heterologous vaccine based on heat-inactivated *M. bovis* (HIMB)^18^.

## Results

### Cell-mediated immune responses after vaccination and challenge

**Figure 1:**
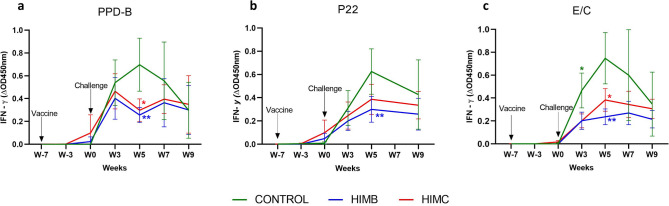
Whole blood IFN-γ responses after vaccination and challenge: The graph shows the IFN-γ levels measured by ELISA. Results are expressed as the increase in optical density (ΔOD 450 mn ± 95% CI). (**a**) Response against bovine tuberculin (PPD-B). (**b**) Response to the P22 complex. (**c**) Response against E/C antigen cocktail. *P < 0.05, **P < 0.01, Kruskal Wallis test with post hoc Dunn’s test. Groups: Control (n = 7, green), HIMB (n = 7, blue), HIMC (n = 7, red). Two animals of the control group were humanely sacrificed at week 7.

The mean antigen-specific whole blood IFN-γ responses before and after challenge for each treatment group are depicted in Fig. 1. A mild increase of IFN-γ responses to PPD-B and P22 were detected in the HIMC vaccinated group at seven weeks after vaccination (Week 0, Fig. 1a,b) although without significant difference when compared to other groups. After challenge, the mean levels of IFN-γ began to increase in the three experimental groups. At week 5, PPD-B specific IFN-γ responses were significantly higher in unvaccinated animals compared to HIMC and HIMB groups (*P* < 0.05 and *P* < 0.01, respectively Fig. 1a). At the same time point, both vaccinated groups also showed lower P22 specific IFN-γ responses compared to the unvaccinated group although they were only statistically significant in the HIMB vaccinated group (*P* < 0.01, Fig. 1b). None of the vaccinated animals showed detectable IFN-γ responses to E/C after vaccination (Fig. 1c). After challenge, the vaccinated groups also showed lower E/C-specific IFN-γ responses than the control group, being statistically significant at weeks 3 (*P* < 0.05) and 5 (*P* > 0.05 and *P* < 0.01 in HIMC and HIMB groups*,* respectively).

**Figure 2:**
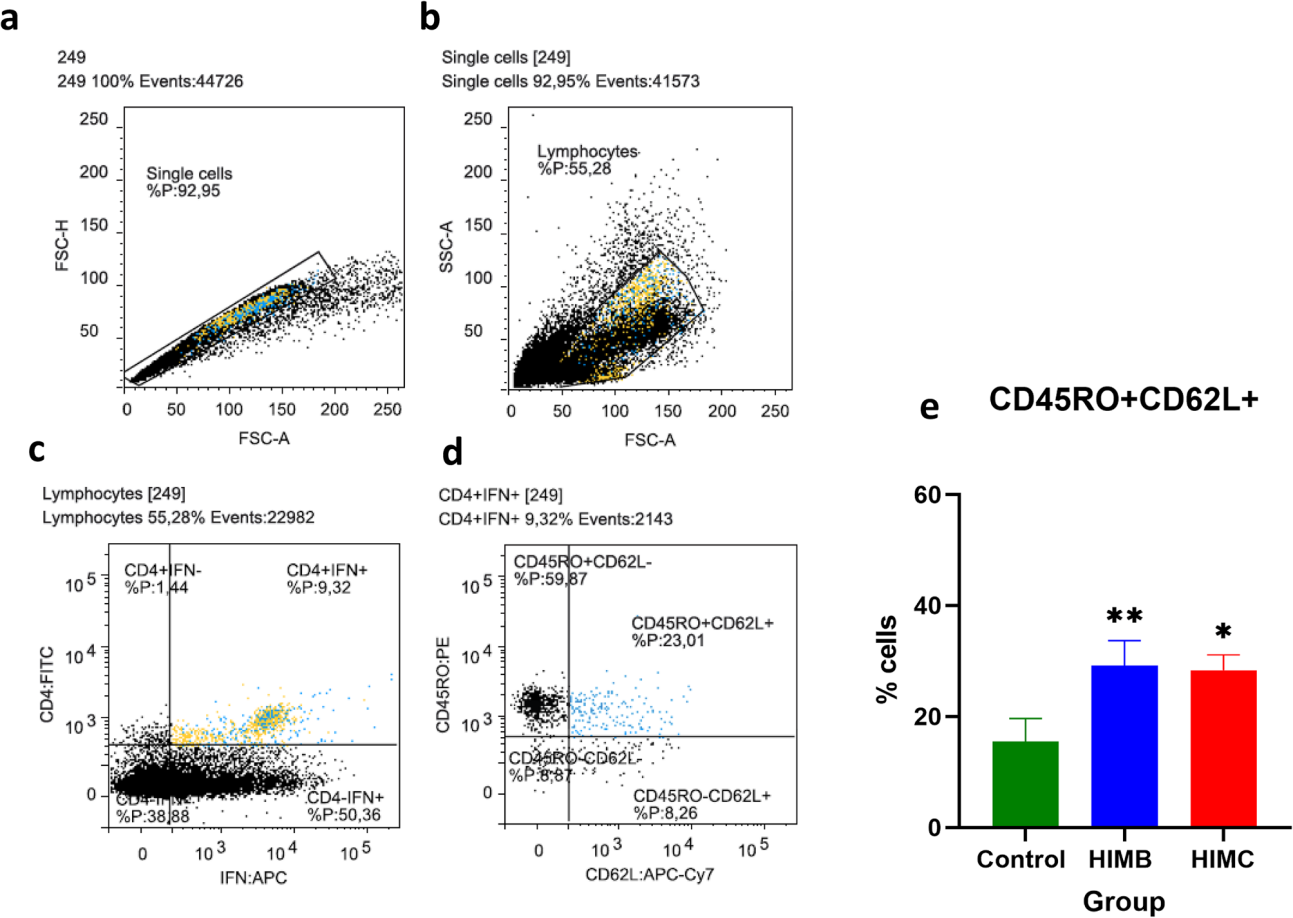
Flow cytometry analysis of IFN-γ producing lymphocytes before *M. caprae* challenge. The analysis was conducted with peripheral blood mononuclear cells (PBMC) isolated from all goats at week 0 (7 weeks post-vaccination and before *M. caprae* challenge) after stimulation with PPD-B overnight. (**a**–**d**) Representative plots of gating strategies. (**a**) Singlet cells identified by forward scatter (FSC). (**b**) Lymphocytes determined by side scatter (SSC). (**c**) Representative frequencies of CD4 cells and intracellular IFN-γ staining (IFNG^+^). (**d**) Representative frequencies of the CD45RO/CD62L subsets gated from CD4 + IFN-γ + cells. (**e**) Frequencies (%) ± 95% CI of CD45RO^+^ CD62L^+^ within the CD4 + IFN-γ-producing cells. Blue dots are the CD4^+^ IFNG^+^ CF45RO^+^ CD62L^+^ cells and the yellow dots represent the rest of CD4^+^ IFNG^+^ cells. Groups: Control (N = 5, green), HIMB (N = 7, blue), HIMC (N = 5, red). *P < 0.05, **P < 0.01, Kruskal Wallis test with post hoc Dunn’s test.

Analysis of IFN-γ-producing cell subsets was performed by flow cytometry at seven weeks post-vaccination (Week 0) using PBMC isolated from the seven HIMB vaccinated, 5 HIMC vaccinated and 5 unvaccinated animals (4 animals did not show sufficient viable PMBC for the assay). Gating strategy is shown in Fig. 2a–d. Both vaccinated groups showed statistically significant higher frequencies of IFN-γ-producing CD4 lymphocytes with a central memory phenotype (C45RO^+^ CD62L^+^) compared to unvaccinated animals (HIMB: *P* < 0.01, HIMC: *P* < 0.05, Fig. 2e).

**Figure 3:**
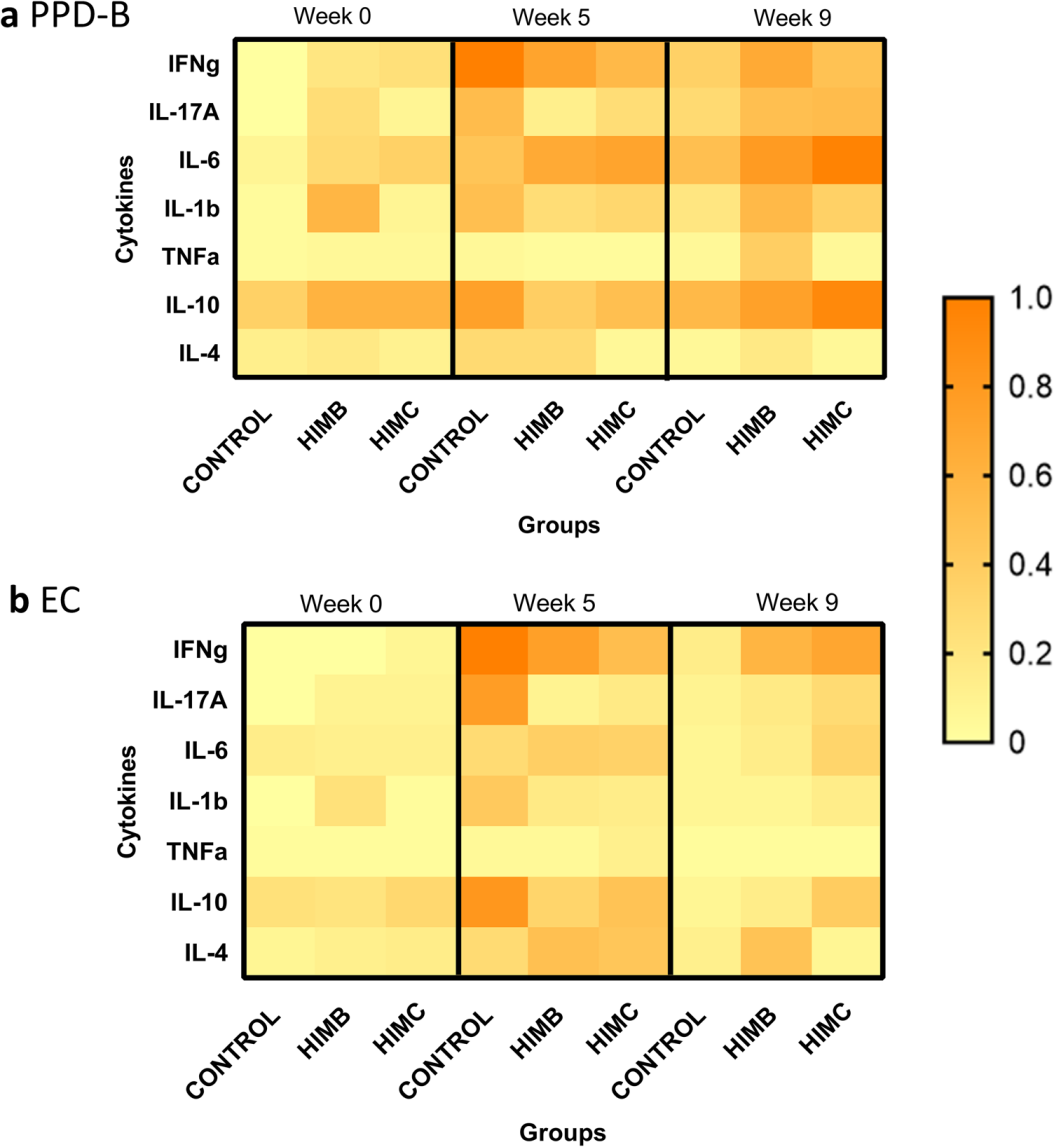
Cytokine profiles before and after *M. caprae* challenge. Cytokine productions detected in plasma after stimulating whole blood with mycobacterial antigens in each experimental group at week 0 (before challenge), and weeks 5 and 9 after challenge. (**a**) *M. bovis* tuberculin (PPD-B). (**b**) ESAT-6/CFP-10 antigen cocktail (E/C). The heat-map represents the mean cytokine production relative to the maximum production (100%) of each cytokine.

Finally, specific production of multiple cytokines after stimulation of whole blood with PPD-B and E/C was analysed at week 0 (before challenge) and at weeks 5 and 9 post-challenge. Results of cytokine production profiles relative to maximum value of each cytokine are presented in Fig. 3. Before challenge (week 0, 7 weeks post-vaccination), both vaccinated groups showed a slightly higher PPD-B-specific proinflammatory (IFN-γ, IL-17A and IL-6) responses compared to the control group (Fig. 3a). Productions of IL-1β in both PPD-B and E/C stimulated blood samples were higher in the HIMB group (*P* < 0.001).

At five weeks after challenge, unvaccinated animals experienced an increase of both proinflammatory cytokines (IFN-γ, IL-17A, IL-1β) and IL-10 production. However, overall cytokine responses decreased in the control group at week 9, when HIMC vaccinated goats showed higher E/C-specific levels of IL-6 compared to other groups (*P* < 0.05, Fig. 3b), and HIMB group showed significantly higher levels of IL-1b to PPD-B (*P* < 0.05, Fig. 3a). At this time point, vaccinated groups also showed a mild increase of both E/C-specific IFN-γ and anti-inflammatory responses (IL-10 and IL-4 in HIMC and HIMB groups, respectively), although not statistically significant (Fig. 3b). No statistically significant differences in TNFα production were observed among groups throughout the experiment.

### Humoral responses after vaccination and challenge

**Figure 4:**
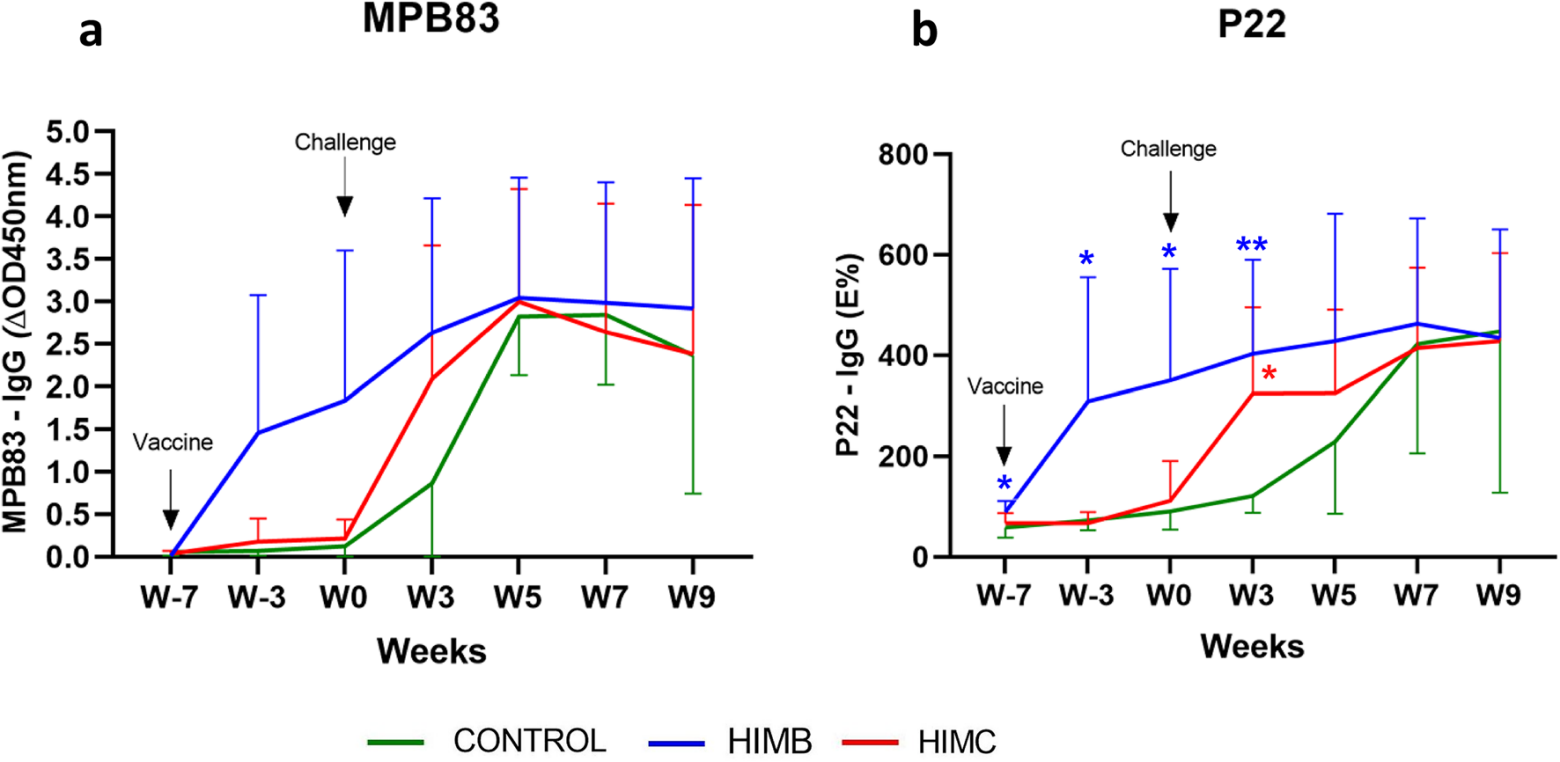
Antibody responses after vaccination and challenge. The figure shows the levels of IgG against MPB83 and P22 antigen measured by ELISA throughout the study. MPB83 results are expressed as the increase of optical density (ΔOD450mn ± 95% CI). P22 ELISA results are expressed as E%: [mean OD450nm of antigen-coated well/(2 × mean negative control OD450nm)] × 100. Groups: Control (N = 7, green, N = 5 at week 9), HIMB (N = 7, blue), HIMC (n = 7, red). *P < 0.05, **P < 0.01, Kruskal-Wallis test with Dunn’s post hoc test.

IgG levels to the antigen MPB83 (Fig. 4a) and the P22 antigenic complex (Fig. 4b) were measured by two ELISAs throughout the study showing similar kinetics at 3 and 7 weeks postvaccination (p.v). HIMB-vaccinated group showed an increase of IgG levels to both antigens compared to HIMC and control groups (Fig. 4), although this increase was only statistically significant in the P22 ELISA (*P* < 0.05, Fig. 4b). Only two HIMC vaccinated animals showed mild IgG responses after vaccination. By contrast, the IgG responses to both antigens increased at 3 weeks post-challenge in both vaccinated groups compared to the control group, although it was only statistically significant in the P22 ELISA (*P* < 0.01 and *P* < 0.05, for HIMB and HIMC groups, respectively), that showed a slightly delayed seroconversion. From week 5 onwards, no significant differences between groups were observed (Fig. 4).

### Clinical signs and body condition after challenge

Animals were observed for clinical signs of TB after *M. caprae* challenge. Results are summarized in Table 1. Mild clinical signs appeared in most non-vaccinated animals at week 3 and evolved to severe clinical signs in all animals at week 7 (two of them were humanely euthanized because end point criteria were reached). Only a few vaccinated animals showed mild clinical signs at week 3 (one of the HIMC and two of the HIMB groups). At the end of the experiment almost all animals developed TB clinical signs. The most observed were cough, dyspnoea, eye discharge, enlarged lymph nodes, runny nose and anorexia.

**Table 1:** Number of animals with clinical signs after *M. caprae* challenge in each treatment group (N = 7).

Group	Week 0	Week 3	Week 5	Week 7	Week 9
CONTROL	0/7	6/7	6/7	7/7	5/5*
HIMB	0/7	2/7	5/7	5/7	6/7
HIMC	0/7	1/7	5/7	7/7	7/7

*At week 7, two animals from the control group were humanely euthanized.

**Figure 5:**
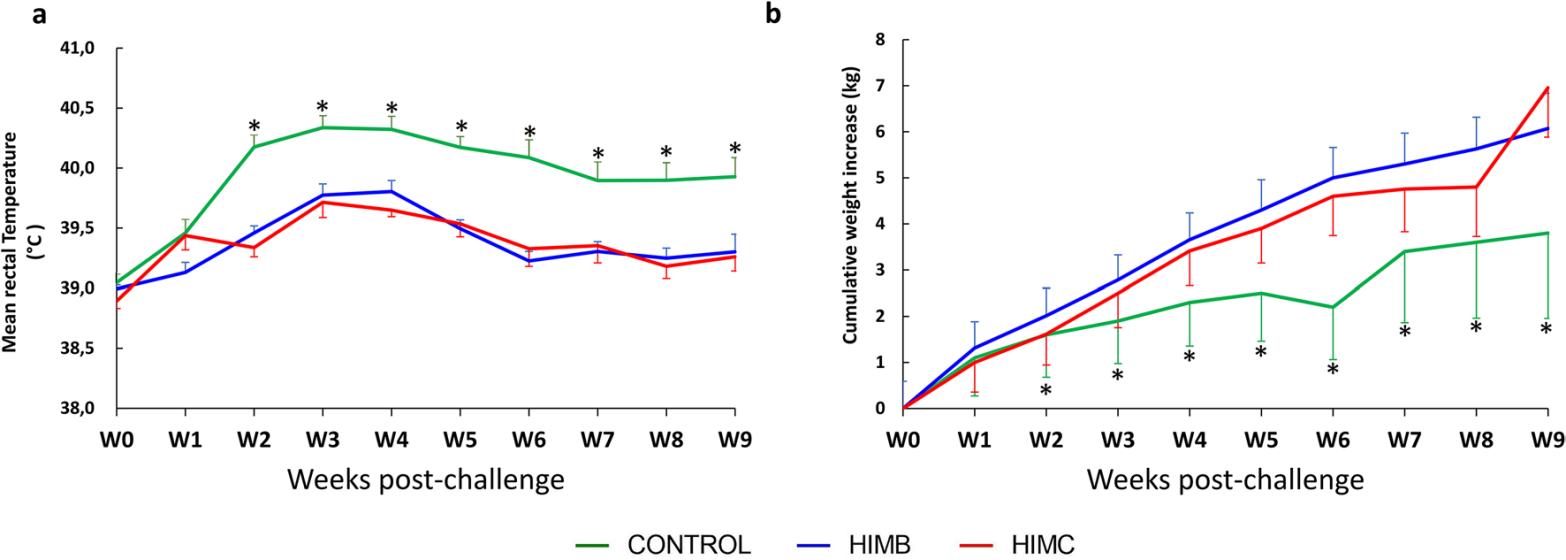
Rectal temperature and body weight. (**a**) Weekly mean rectal temperature of each group expressed in Celsius degrees (°C ± 95% CI). (**b**) Weekly mean cumulative body weight increases of each group expressed in ΔKg (Kg at each week post-challenge minus Kg at week 0) ± 95% CI. Groups: Unvaccinated control (green, N = 7 and N = 5 at weeks 8 and 9), vaccinated with HIMB (blue, N = 7) and vaccinated with HIMC (red, N = 7). *P < 0.05 between control and both vaccinated groups, one-way ANOVA with Tukey test for multiple pairwise comparisons.

Rectal temperature and body weight were measured weekly after challenge (Fig. 5). The mean rectal temperature of the vaccinated groups was significantly lower than the control group from week 2 onwards (*P* < 0.05, Fig. 5a). No significant differences were observed between vaccinated groups. The mean body weight gain was significantly higher in vaccinated groups compared to the control group from week 3 onwards (*P* < 0.05, Fig. 5b). At the end of the experiment, the mean cumulative weight increase of the control group was 2.3 kg (− 0.9 to 5.4, 95% CI), significantly lower (*P* < 0.05) than the vaccinated groups (HIMB: 6.1 kg, 95% CI 5–7.7; HIMC: 7.0 kg, 95% CI 1.6–12.4). Again, no statistically significant differences were observed between HIMB and HIMC groups throughout the experiment.

### Post-mortem findings

**Figure 6:**
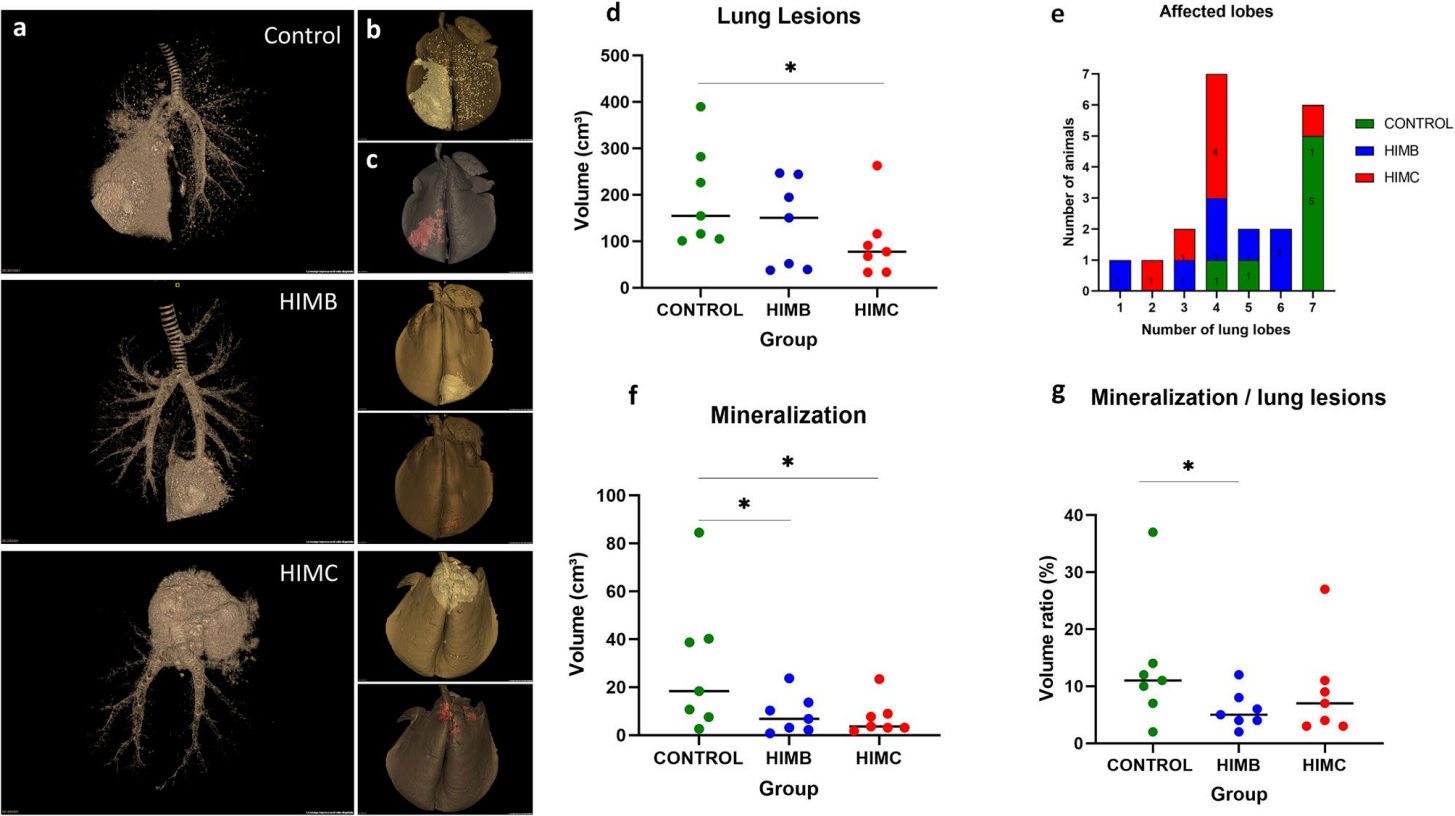
Lung pathology assessed by computed tomography (CT) of intratracheally formalin perfused lungs. Example of the images obtained from the lungs of an unvaccinated control and two animals vaccinated with HIMB and HIMC, respectively. (**a**) Larger images on the left show the tracheobronchial tree and the TB lesions. (**b**) The top right miniatures show the TB lesion (whitish) superimposed onto the lung volume (yellowish). (**c**) The bottom right miniatures show (in red) the mineralized portion of the TB lesion. (**d**) Volumes of lung TB lesions in each group expressed in cm^3^. (**e**) To evaluate the lesion dispersion within the lungs in each group, the number of lobes with TB lesions in each animal is plotted against the number of animals. The control group has a higher number of animals with a higher number of affected lobes than the vaccinated groups. (**f**) Volume of lesion mineralization expressed in cm^3^. (**g**) Ratio between the volume of mineralization and the volume of lung lesions expressed in %. Horizontal lines in (**d**), (**f**) and (**g**) represent the median values. Groups: Control (n = 7, green), HIMB (n = 7, blue), HIMC (n = 7, red). *P < 0.05, **P < 0.01, Kruskal–Wallis test followed by one-tailed Dunn’s test for multiple comparisons.

All goats showed TB lung lesions at necropsy. The assessment of lung lesions using CT is shown in Fig. 6. HIMC vaccinated group showed lower median values of the lung lesions volumes (78 cm^3^, 95% CI: 33–263) compared to the control group (155 cm^3^, 95% CI: 101–390; *P* < 0.05), and HIMB vaccinated group (151 cm^3^, 95% CI: 38–247; yet not statistically significant, Fig. 6d). Vaccinated groups showed lower intrapulmonary TB dissemination assessed by mean number of affected lobes (HIMC: 4.0, 95% CI 2.6–5.4, *P* < 0.01; HIMB: 4.1, 95% CI 2.5–5.8, *P* < 0.05) compared to the control group (6.3, 95% CI 5.1–7.4). Five out of seven unvaccinated animals showed TB lesions in the seven lung lobes whereas 6/7 HIMC and 4/7 HIMB vaccinated animals showed four or less lung lobes affected (Fig. 6e). In addition, vaccinated groups showed significantly lower median values of lung mineralization volumes (HIMC: 3.6 cm^3^, 95% CI: 1.8–23.4; HIMB: 6.8 cm^3^, 95% CI 0.8–23.7) compared to the control group (18.4 cm^3^, 95% CI 2.6–84.5, *P* < 0.05, Fig. 6f), and lower mineralization volume ratios of lung lesions (HIMB: 5%, 95% CI 2–12, *P* < 0.05; HIMC: 7%, 95% CI 3–27; Control: 11%, 95% CI 2–37; Fig. 6g).

**Figure 7:**
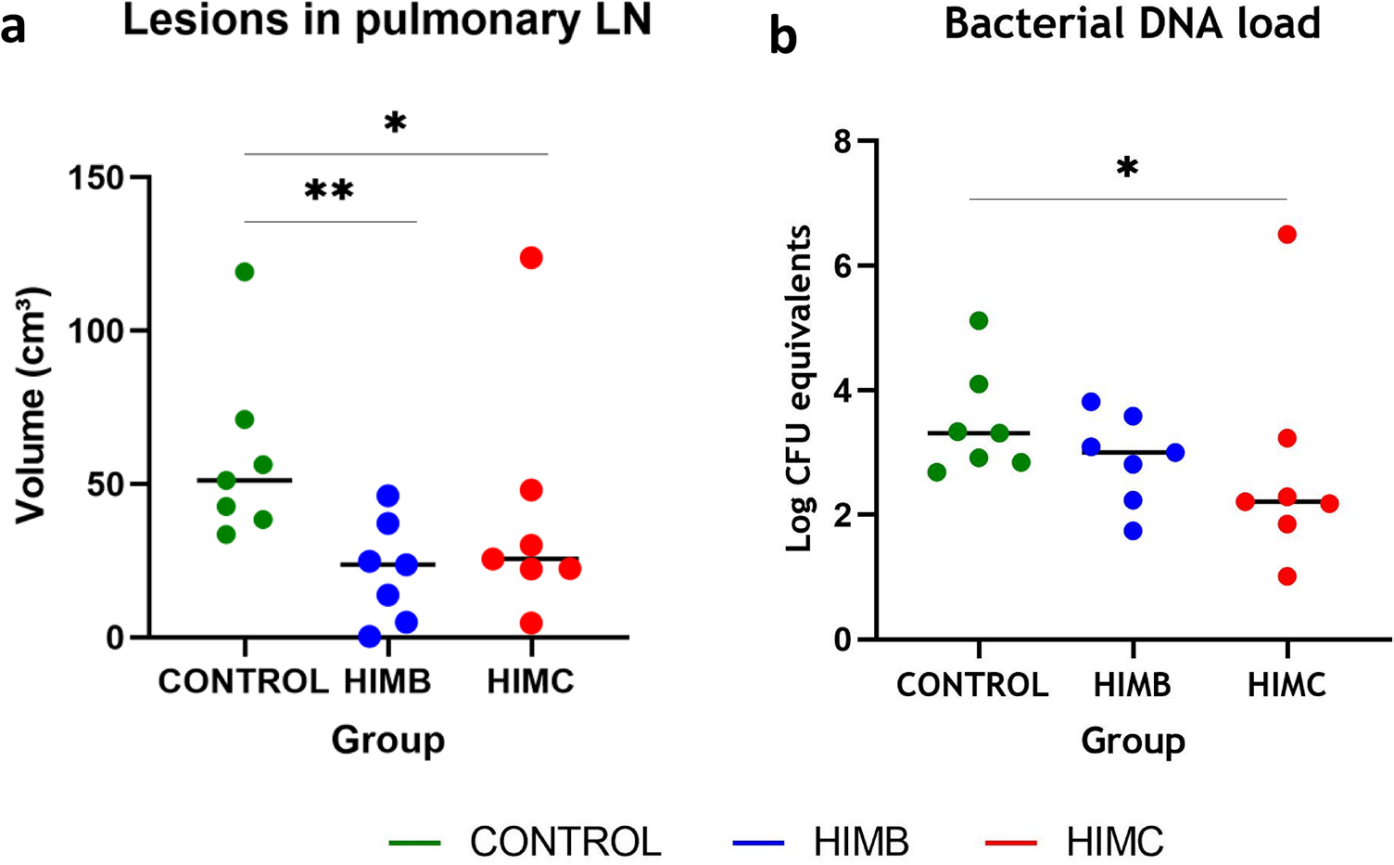
Post-mortem results in pulmonary lymph nodes (LN). (**a**) Volume of lesions in LN expressed in cm^3^. (**b**) *M. caprae* DNA load in LN measured by quantitative PCR and expressed as Log_10_ CFU equivalents. *P < 0.05, **P < 0.01, Kruskal–Wallis with post hoc Dunn tests.

In pulmonary LN, the median of TB lesions volumes was also significantly higher in the unvaccinated group (51.2 cm^3^, 33.6–119.2, 95% CI) compared to HIMB (23.7 cm^3^, 0.3–46.2, 95% CI; *P* < 0.01) and HIMC (25.6 cm^3^, 4.7–123.8, 95% CI; *P* < 0.05) groups (Fig. 7a). The bacterial DNA load in pulmonary LN, as estimated by qPCR, was significantly lower in HIMC vaccinated animals (*P* < 0.05) and slightly lower (but not statistically significant) in HIMB vaccinated animals compared to the control group (Fig. 7b).

With regard to extrapulmonary TB, the control group was the only one with all animals showing extrapulmonary lesions, a proportion significantly higher to that found in vaccinated groups (4/7 each, *P* < 0.05). A summary of extrapulmonary lesions found in each treatment group and their location is shown in Table 2.

**Table 2:** Number of animals with extrapulmonary TB lesions and their location in each treatment group (N = 7).

	Retropharyngeal LN	Sub-mandibular LN	Mesenteric LN	Hepatic LN	Liver	Spleen	Total extra-pulmonary lesions
Control	2/7	1/7	4/7	3/7	1/7	7/7	7/7
HIMB	1/7	0/7	0/7**	1/7	0/7	3/7**	4/7*
HIMC	2/7	0/7	1/7*	1/7	1/7	1/7***	4/7*

*P < 0.05, **P < 0.01, ***P < 0.001, Chi-square test.

### Cross-sectional analysis: biomarkers of vaccine efficacy

To assess immunological markers as predictors of vaccination outcome, the antigen-specific IFN-γ-producing CD4 central memory T (TCM) cell subsets (CD45RO^+^ CD62L^+^) obtained by flow cytometry in each vaccinated animal (N = 12) were correlated with clinical, pathological and bacteriological results of the same animal (Table 3). Results showed significant inverse correlations of TCM % with volume of lung lesions and lesion volume / lung volume ratio, whereas no correlation was observed with LN lesions and bacterial DNA load in LN. Regarding clinical signs, body weight increase after challenge (mean Kg at week 9 minus mean Kg at week 0) showed significant direct correlation with TCM % and mean rectal temperature obtained at the temperature peak time point (week 3) showed significant inverse correlation with TCM %.

**Table 3:** Correlation between vaccine-induced immunity and vaccination outcome.

	Vol. lung lesions	Vol. lesions/vol. lung	Vol. LN lesions	DNA load in LN	Weight increase	Peak temperature
	(cm^3^)	(%)	(cm^3^)	(Log_10_ CFUeq)	(ΔKg)^b^	(°C)^c^
TCM (%) ^a^	− 0.75** (− 0.93 to − 0.29)	− 0.85*** (− 0.96 to − 0.54)	0.22 (− 0.45 to 0.71)	− 0.07 (− 0.63 to 0.54)	0.54* (− 0.07 to 0.86)	− 0.61* (− 0.88 to − 0.04)

Values show Spearman rank coefficient ± 95% CI.

*Vol.* volume, *LN* lymph nodes, *CFUeq* colony forming units equivalents.

*P < 0.05; **P < 0.01; ***P < 0.001.

^a^IFN-γ-producing CD4 central memory T-cells at week 0

^b^Week 9–week 0

^c^Week 3.

## Discussion

Inactivated mycobacterial vaccines can be adapted ad hoc to field conditions by generating autovaccines with the same strains circulating in the herd or in a specific outbreak area. This is the first proof-of-concept study to evaluate the efficacy of a heat-inactivated autovaccine against *M. caprae* infection in goats.

Nowadays, there is a certain degree of background regarding the inactivated vaccine candidate based on *M. bovis* (HIMB), that have been reported to protect goats against TB in laboratory and field conditions^18,20^, as well as cattle^21^, wild boar^22,23^, red deer^24^, badgers^25^ and wild pigs^26^. HIMB represents an interesting alternative to live-attenuated BCG vaccine since the deployment logistics are simpler and the strain survival is not required^27^. In the present study, the gross pathology and bacteriological results, as well as the clinical follow-up after experimental challenge with *M. caprae*, ratified that MTBC-inactivated vaccines conferred protection when compared to unvaccinated animals.

Moreover, the HIMC group, vaccinated with the *M. caprae* strain homologous to the challenge strain (autovaccine proof-of-concept), showed slightly better protection against pulmonary TB compared to the group vaccinated with the heterologous vaccine (HIMB), with lower volume of lung lesions and animals showing lower number of affected lung lobes. In addition, the HIMC vaccinated group presented significantly lower bacterial DNA load in pulmonary LN, even when considering that one of the animals vaccinated with HIMC showed a huge amount of bacterial load (over 10^6^ CFU equivalents, very much higher than any unvaccinated goat). This animal also showed very high pathological extension compared to the rest of the group (being an outlier for all parameters). Intriguingly, this goat did not show detectable immune responses (neither to ELISA nor IGRA) after vaccination. Even though an unnoticed incorrect administration of the vaccine cannot be ruled out, this animal could have been immunocompromised at the vaccination time point, and thus would have not been properly immunized.

Interestingly, vaccinated groups not only did show a reduced volume of lung lesions and lung mineralization compared to the control group, but also showed lower volume ratio of mineralization within the lung lesions, suggesting that the lung pathology was in earlier/more contained developmental stages compared to unvaccinated animals according to criteria previously described for *M. bovis* infected cattle using histopathology^28^. This approach defined four granuloma developmental stages according to different parameters such as the degree of mineralization. Using these criteria, cattle vaccinated with BCG or BCG-prime / heterologous boost regimes also showed lower proportion of lung lesions in advanced developmental stages^29,30^.

Vaccination also reduced the incidence and spread of extrapulmonary TB as previously described in BCG or HIMB vaccinated goats^18^. All unvaccinated animals showed TB lesions in the spleen and, in this control group there was also a higher number of animals with lesions in hepatic and mesenteric LN, indicative of a reduced lymphohematogenous dissemination of mycobacteria in both vaccinated groups.

Comparisons of clinical parameters, particularly the body weight increase and rectal temperature kinetics, were very consistent with post-mortem results. The body weight change and the rectal temperature at 3 to 5 weeks post-challenge are useful indicators of vaccination outcome as previously demonstrated in experimental trials conducted in goats and sheep^18,31,32^, and should be considered as valuable parameters in experiments to assess the efficacy of vaccines and treatments against TB.

Even though the administration of inactivated vaccines induced detectable ex vivo IFN-γ responses to PPD-B and P22 using IGRAs, in a number of animals, no responses were detected when using an E/C protein cocktail after vaccination and before challenge. This result agrees to previously reported in HIMB parenterally vaccinated goats^18^. In addition, the intensity of E/C-specific IFN-γ responses after challenge were equivalent to that induced by PPD-B and P22, indicating that this cocktail, initially developed for TB diagnosis and compatible with BCG vaccination, could be also suitable as a DIVA reagent for IGRA tests after administration of MTBC inactivated vaccine.

Unvaccinated animals showed higher IFN-γ levels measured by the IGRAs after challenge compared to vaccinated ones, reaching a peak response at five weeks post-challenge. This trend was confirmed by increased proinflammatory responses in unvaccinated animals measured by the multi-cytokine assays performed at 5 weeks post challenge. At this time point, unvaccinated group showed higher antigen-specific IFN-γ, IL-17A and IL-1β levels compared to vaccinated groups. This multi-cytokine proinflammatory pattern has also been described in buffalos infected with *M. bovis*^33^ that showed increased proinflammatory cytokines and chemokines (IP-10, IFN-γ, IL-6, IL-17A, MIP-1β, MIP-1α and MCP-1) in whole blood stimulated with the E/C whereas anti-inflammatory cytokines (IL-4 and IL-10) remained in basal levels. On the contrary, in the present experiment unvaccinated goats also showed increased IL-10 responses at 5 weeks post challenge. The anti-inflammatory effect induced by this increase of IL-10 levels in unvaccinated goats could be associated to the subsequent decrease of proinflammatory responses (to both IGRA and multi-cytokine assays at week 9 post vaccination), while vaccinated animals maintained moderate levels throughout the experiment.

In this study, the contained IFN-γ responses in vaccinated animals agreed with a more favourable disease outcome. However, whole blood ex vivo IFN-γ responses in vaccine trials should be interpreted with caution since previous studies reported that BCG vaccinated goats showed reduced^34^, similar^35^ or even higher^18^ antigen-specific IFN-γ responses after *M. caprae* challenge compared to unvaccinated controls.

Therefore, more accurate immunomarkers are required as predictors of vaccine efficacy and correlators of disease progression. The most useful immunological biomarkers to predict the vaccine efficacy are those that can be measured before the infection/exposure of animals to mycobacteria. With this purpose, as well as to estimate the duration of the vaccine-induced immunity, we assessed the proportion of antigen-specific IFN-γ-producing TCM subsets, based on the expression of CD45RO and CD62L receptors^36^.

Individual PPD-B-specific IFN-γ-producing TCM % correlated with favorable disease outcome parameters, namely, reduced lung lesions, higher body weight gain, and lower peak of rectal temperature, suggesting that this immunological parameter can be a useful biomarker to infer the vaccine-induced protective immunity. Similarly, proliferation of antigen-specific memory T-cells determined by cultured IFN-γ ELISPOT were also inversely associated to the pathology degree in vaccinated cattle and goats^35,37^.

On the other hand, the expansion of cells with a TCM phenotype elicited by vaccination is suggestive of long-term immunity as reported for mice and humans^36,38^. Nevertheless, the duration of the vaccine-induced immunity cannot be properly estimated in merely a seven-week period after vaccination and longer studies are required to confirm the results. In long-term trials, a wane of BCG-induced ex vivo IFN-γ response has been observed in goats from 24 weeks p.v. onwards^9^ and, approximately one year after vaccination, the proportion of PPD-B-specific IFN-γ-producing T-memory cells (CD45RO+) was similar to that of unvaccinated goats, although it increased significantly when measured three weeks after revaccination^32^.

Finally, serological results confirm the ability of inactivated vaccines, particularly HIMB, to induce MTBC-specific antibody responses^18^ and, as such, the specificity of the serology will be compromised as a TB diagnostic tool in vaccinated animals^39^. Surprisingly, the seroconversion was less frequent and less intense (when it occurred) after HIMC vaccination. A lower expression of MPB83 (also a major component of P22 reagent) or a lower exposure of this protein in the *M. caprae* cell surface compared to *M. bovis* might be explanatory factors of this different antibody-response patterns, although the underlaying mechanisms were not investigated and further comprehensive and focused studies should be carried out to confirm this hypothesis.

Given that autovaccines may have advantages, particularly from the regulatory point of view, and that heat-inactivated mycobacterial vaccines are easy to produce, the results of this proof-of concept study encourage to conduct further autovaccine field trials in natural infection settings to validate this vaccination strategy that can contribute to control TB in goats in high-prevalent scenarios when test-and-cull is unfeasible.

## Methods

### Animals, vaccination schedule and experimental infection

Twenty-one female Pyrenean breed goats of approximately 2 months of age, from an officially TB-free herd located at the Catalan Pyrenees (Spain) were divided into 3 experimental groups of 7 animals each: Unvaccinated control group, group vaccinated with heat-inactivated *M. bovis* (HIMB) and group vaccinated with heat-inactivated *M. caprae* (HIMC).

Inactivated vaccines were produced at NEIKER (Basque Country, Spain) and prepared as previously described^22^. Briefly, HIMB was prepared from a *M. bovis* field strain (SB0339,www.Mbovis.org) isolated from a naturally infected wild boar, while HIMC was produced by the same procedure using a *M. caprae* field strain (SB0157, www.Mbovis.org) isolated from a tuberculous sheep in Catalonia^40^. Both field strains were sub-cultured in Middlebrook 7H9 broth (BD Diagnostics, USA) enriched with Oleic acid-Albumin-Dextrose-Catalase (OADC) for 3 weeks approximately and subsequently washed, tittered and inactivated as previously described^18^. Inactivated mycobacterial suspensions were oil-adjuvanted with Montanide ISA 61 VG (Seppic, Paris, France) as indicated by the manufacturer. Final concentration of vaccines was approximately 5 × 10^7^ inactivated CFU/ml. All animals were subcutaneously vaccinated (on the right scapula) with 1 ml of HIMB or HIMC.

Six weeks after vaccination, all experimental animals were transferred to the Biosafety Level-3 (BSL-3) facility of IRTA-CReSA (Catalonia, Spain) and were housed in two experimental boxes with 3–4–4 and 4–3–3 animals from Control, HIMB and HIMC groups in each box, respectively. Animals were fed with hay, alfalfa, mineral salt and maintained with water ad libitum throughout the experiment.

After a week of acclimatation, the animals were sedated with an intramuscular administration of acepromazine maleate (0.05 mg/kg) and butorphanol (0.2 mg/kg) and subsequently anesthetized with the intravenous administration of propophol (5 mg/kg) and midazolam (0.2 mg/kg). Afterwards, the animals were endonbronchially challenged as previously described^2^ with 0.5 ml of an approximately 2 × 10^3^ CFU/ml suspension of the same *M. caprae* strain used to prepare the HIMC vaccine.

After challenge, the animals were daily monitored for clinical signs. Rectal temperature and body weight were measured every week. Animals were bled at weeks -7 (vaccination time point), -3, 0 (challenge time point), 3, 5, 7 and 9 (end point) for immunological assays.

The experimental design and the experiments were conducted following the recommendations in ARRIVE guidelines (https://arriveguidelines.org/).

### Ethics statement

All procedures involving experimental animals carried out during the study followed the recommendations in the ARRIVE guidelines (https://arriveguidelines.org/), and were approved by the Animal Welfare Committee of the *Generalitat de Catalunya* (Project No. 10794) in conformity with relevant guidelines and regulations and the European Union legislation for the protection of experimental animals (86/609/EEC, 91/628/EEC, 92/65/EEC and 90/425/EEC).

### Antigens and reagents

*Mycobacterium bovis* tuberculin (PPD-B, 2500 IU/ml) was obtained from CZ Vaccines (Porriño, Spain). The recombinant MTBC-specific antigens ESAT-6, CFP-10 and MPB83 were obtained from Lionex (Braunschweig, Germany) at a concentration of 500 μg/ml each. ESAT-6 and CFP-10 were mixed 1:1 in an antigenic cocktail (E/C). The antigenic complex P22 was produced by immunopurification of PPD-B (CZ Vaccines) as previously described^41^ and supplied by the *Instituto de Investigación Carlos III* (Madrid, Spain), at a concentration of 500 μg/ml.

### Ex vivo IFN-γ release assay (IGRA)

Whole blood samples were collected at time points mentioned above from the jugular vein using heparinized tubes and subsequently stimulated in 96-well cell culture plates (Eppendorf, Hamburg, Germany) with PPD-B, P22 (except at week 7) and E/C at a final concentration of 20 μg/ml. Phosphate-buffered saline was used as an unstimulated control. Samples were incubated at 37 ºC and 5% CO_2_ for 18 ± 2 h. Plasma supernatants were collected after centrifugation at 18 g for 10 min and the released IFN-γ was measured by ELISA according to the ID.screen® Ruminant IFN-g kit (ID.vet, Grabels, France) instructions. ELISA plates were read as optical density obtained at 450 nm (OD_450nm_) using a spectrophotometer (Biotek Power Wave XS®, Agilent, dana Clara, USA). Antigen-specific IFN-γ responses were calculated as ΔOD_450nm_ of antigen-stimulated well minus OD_450nm_ of non-stimulated well (ΔOD_450nm_).

### Multi-cytokine quantification assay

Cytokines in plasma supernatants from PPD-B and E/C stimulated and unstimulated whole blood samples were quantified at weeks 0, 5 and 9 post-challenge using a bovine customized multiplex assay kit (MILLIPLEX® Millipore, Merck Life Science S.L.U., Madrid, Spain) including a seven-plex panel for IL-1β, TNFα, IL-6, IL-10, IL-4, IFN-γ and IL-17A. 25 μl of each plasma sample and cytokine standards were analysed following the manufacturer’s instructions using xMAP® technology (Thermofisher Scientific, Waltham, MA, USA). ELISA plates were read on MAGPIX instrumental platform with xPONENT acquisition software (Thermofisher Scientific).

### Flow cytometry

At week 0 (before challenge), peripheral blood mononuclear cells (PBMC) were isolated from blood samples and stimulated for 16 h (2 × 10^6^ cells/well) with PPD-B (10 μg/ml) as previously described^32^. At week 0, 2 × 10^5^ cells were stained with monoclonal antibodies (mAb) that recognise bovine IFN-γ (anti-bovine CC302 IgG1-Alexa Fluor® 647) from Bio-Rad Laboratories Inc. (Hercules, CA, USA), CD4 (anti-sheep 44.38 IgG2A-FITC), CD45RO (anti-bovine IL-A116 IgG3-RPE) and CD62L (anti-bovine CC32 IgG1-APC-Cy7) as previously described^32^. Stained cells were re-suspended in 200 µl PBS with 1% paraformaldehyde and analysed by flow cytometry in FacsARIA II (BD Diagnostics, Sparks, USA). Data were analyzed using FlowLogic TM from Inivai TM Technologies (Mentone, Victoria, Australia).

### Antibody detection assays

Plasma samples from all experimental animals were analysed in duplicate to follow-up the antibody responses to MTBC after vaccination and challenge. Two indirect ELISAs were used to detect total IgG against the MPB83 antigen and the P22 antigenic complex, respectively. The two ELISAs were performed as previously described^42,43^. MPB83-IgG levels were calculated as OD450nm of antigen-coated well minus OD450nm of non-coated well (ΔOD450nm), whereas P22-IgG levels were calculated as ELISA percentage (E%) = [mean OD450nm of antigen-coated well/(2 × mean negative control OD450nm)] × 100.

### Necropsy, pathological examination and computed tomography (CT)

Experimental animals were euthanized at 9 weeks post-challenge with an overdose of intravenous sodium pentobarbital (two goats from the control group were humanely euthanized at week 7). At necropsy, the diameter of TB compatible lesions in retropharyngeal (right and left), mediastinal (cranial and caudal) and tracheobronchial lymph nodes (LN) were measured, and the volume of lesions was inferred as previously described^31^. Other visible lesions in extrapulmonary tissues were also recorded and fixed in 10%-buffered formalin to be confirmed by histopathology and the Ziehl Neelsen stain to detect acid-fast bacilli. Lungs were also formalin-fixed, including airways perfusion with formalin before immersion, as previously described^2^, and then scanned by computed tomography (CT) using a 16-slice multi-detector scanner (Brivo CT-385, General Electric Healthcare, Madrid, Spain). CT analysis was performed as previously described^31^. Briefly, total volumes of lungs and TB lesions were measured using volume rendering images with different density patterns (i.e. solid, mineralized and cavitary lesions), and total volume of mineralization was calculated using 100–300 Hounsfield units.

### Bacterial DNA quantification

After macroscopic pathologic evaluation, the whole mediastinal and tracheobronchial LNs were stored at − 20 °C until they were processed for bacteriology (one month later). Then LNs were thawed and homogenized in 10 ml of sterile distilled water using a homogenizer (Masticator, IUL Instruments, Barcelona, Spain). An aliquot of 1 ml of each homogenate was inactivated at 75 °C for 1 h and, in parallel, an aliquot of 100 ml of the *M. caprae* master seed (~ 10^8^ CFU/ml), used for the challenge inoculum, was also inactivated and then ten-fold serially diluted to establish the standard curve. DNA samples were extracted using the ID Gene™ spin universal extraction kit (ID.vet) and amplified with the *Mycobacterium tuberculosis* complex Duplex kit (ID.vet). Amplification was performed in a 7500 fast real-time PCR system (Applied Biosystems, Walham, MA, USA). CFU genomic equivalents were calculated as previously described^44^.

### Data analysis

A completely randomized design was carried out for studying the effects of vaccinations as a primary factor. Temperature and body weight were compared by using unidirectional ANOVA followed by Tukey test for multiple comparisons.

Antigen-specific immune responses (measured by IGRA, IgG-ELISA, Multi-cytokine assay and Flow Cytometry) were compared by using Kruskal–Wallis test with post hoc Dunn’s test. Volumes of lesions in LN and lungs (measured by direct visual inspection and CT, respectively), and mycobacterial DNA load (measured by qPCR) were compared by using Kruskal–Wallis test with post hoc one-tailed Dunn’s test.

Correlations of flow cytometry results (% of antigen-specific IFN-γ-producing T-memory cell subsets) with clinical and post-mortem parameters were assessed by using one-tailed non-parametric Spearman's Rank correlation coefficient. A *p*-value less than 0.05 was considered statistically significant.

GraphPad Prism version 8.0.0 (San Diego, CA, USA) was used for the statistical analysis.

## Data Availability

The databases generated and analyzed during the current study are available from the corresponding author on a reasonable request.
